# Timing of rabbit antithymocyte globulin induction therapy in kidney transplantation: an observational cohort study

**DOI:** 10.1186/2047-1440-3-1

**Published:** 2014-01-03

**Authors:** Jennifer J Harrison, Bassem Hamandi, Yanhong Li, Olusegun Famure, S Joseph Kim

**Affiliations:** 1 Department of Pharmacy Services, Toronto General Hospital University Health Network 585 University Avenue, Munk Building, BCS-076, M5G 2 N2 Toronto, Ontario, Canada; 2 Department of Pharmacy Services, Toronto General Hospital University Health Network 200 Elizabeth Street Eaton Building, EN B-214, M5G 2C4 Toronto, Ontario, Canada; 3 Toronto General Hospital University Health Network 585 University Avenue, 11C-1183, M5G 2 N2 Toronto, Ontario, Canada; 4 Toronto General Hospital University Health Network 585 University Avenue, 11C-1198b, M5G 2 N2 Toronto, Ontario, Canada; 5 Division of Nephrology and the Kidney Transplant Program, Toronto General Hospital University Health Network 585 University Avenue, 11C-1183, M5G 2 N2 Toronto, Ontario, Canada

**Keywords:** Induction therapy, Kidney transplantation, Outcomes, Cohort study

## Abstract

**Background:**

Literature on the timing of rabbit antithymocyte globulin (rATG) induction and its effects on kidney transplant outcomes is limited. The manufacturer recommends that the first dose be given intra-operatively, however this may present clinical practice risks and challenges. Our objective was to assess the impact of the timing of the first dose of rATG on kidney transplant outcomes.

**Methods:**

Incident kidney transplant recipients (KTR) from January 2002 to December 2009 receiving the first dose of rATG post-operatively (Post, n = 353) or before reperfusion (Pre, n = 124) were evaluated. Outcomes assessed included eGFR at 1-year, delta eGFR (12 versus 1 month), and incidence of biopsy-proven acute rejection, graft loss, death, and a composite of the time-to-event outcomes. The impact of timing on outcomes was adjusted for potential confounders and assessed using linear and Cox regression models.

**Results:**

Among 435 KTR surviving with function to 12 months post-transplant, there was no significant difference in mean estimated glomerular filtration rate or eGFR (55.0 versus 56.7 mL/min, *P* = 0.46) and delta eGFR (1.8 versus 0.3 mL/min, *P* = 0.40) in Post versus Pre groups, respectively. At a median follow-up of 3 years, the composite endpoint (time to first biopsy-proven acute rejection, graft loss, or death) was similar by timing group (adjusted HR = 0.94; 95% CI: 0.58, 1.53, *P* = 0.81) in the total study population.

**Conclusions:**

Timing of rATG had no appreciable impact on clinically relevant endpoints in this study cohort. These results support consideration of more flexible timing of the first dose of rATG induction in KTR.

## Background

Rabbit antithymocyte globulin (rATG, Thymoglobulin™, Genzyme Canada Inc., Mississauga, ON, Canada) is a polyclonal gamma immunoglobulin derived from the immunization of rabbits with human thymocytes and indicated for the prevention and treatment of acute kidney transplant rejection [1]. Induction with rATG, together with maintenance immunosuppression (that is, calcineurin inhibitor, anti-metabolite, and corticosteroids), has been shown to be more effective than maintenance immunosuppression alone in preventing episodes of acute rejection in adult kidney transplant recipients (KTR) [2,3]. However, adverse events such as fever, chills and gastrointestinal distress are more frequent with rATG than with other induction agents [4,5]. Serious reactions such as cytokine release syndrome with hemodynamic instability can also occur and are most commonly associated with the first dose and rapid infusion rates [1].

The question of timing of first dose of rATG induction is relevant in clinical practice since operational factors may favor postoperative administration. The manufacturer advises intra-operative dosing, a recommendation supported by one randomized controlled trial in which intra-operative dosing led to a reduction in delayed graft function (DGF) and lower serum creatinine at 14 days post-transplant when compared to postoperative dosing [6]. However, adverse drug events resulting from medication error occur commonly in the peri-operative setting and are a major cause of morbidity and mortality [7].

The operating room environment, with its multiple diversions and production pressures, dynamic changes in patient physiology, and the potential for provider fatigue, all contribute to this safety risk [7]. For this reason, we have implemented a practice change at our center in recent years to begin administration of induction therapy in the immediate postoperative period. There is a paucity of published data to support this approach and little is known about the impact of timing of the initial dose on long-term outcomes.

The primary objective of this study was to compare renal function at one year in KTR receiving the first dose of rATG postoperatively versus prior to graft reperfusion. The secondary objective was to compare the risk of achieving the composite endpoint of biopsy-proven acute rejection (BPAR), graft loss, or death across induction timing groups.

### Patients and methods

#### Study design and population

We conducted a single-center observational cohort study of all *de novo* KTR receiving rATG induction therapy from 1 January 2002 to 31 December 2009 with follow-up until 31 December 2010. We included all patients receiving at least one dose of rATG for the purpose of induction within 7 days of transplantation. Patients who received a partial course of another induction agent (for example, basiliximab) were included. Excluded from the analysis were multi-organ transplant recipients, cases of primary nonfunction, and patients undergoing desensitization protocols.

Standard maintenance immunosuppression during the study period included a calcineurin inhibitor, mycophenolate mofetil, and prednisone. Until 2005, the first-line calcineurin inhibitor was cyclosporine microemulsion, with C_2_ level monitoring to a target of 1,700 ng/mL post-transplant. Subsequently, tacrolimus became the standard calcineurin inhibitor with trough level monitoring to a target of 10 to 15 ng/mL or 5 to 8 ng/mL for high and low immunologic risk patients, respectively. Some patients at high risk of developing new-onset diabetes mellitus were initiated on cyclosporine microemulsion. Since 2005, the initial C_2_ target for cyclosporine microemulsion has been modified to 1,000 to 1,200 ng/mL. Standard steroid therapy included a pre-operative dose of intravenous methylprednisolone 1 mg/kg followed by an oral prednisone taper. Prior to 2006, patients received a slow prednisone taper to 5 mg per day over the first 3 months. Since 2006, all patients at low immunologic risk receive a rapid prednisone taper to 5 mg per day by postoperative day 7. Mycophenolate mofetil was used at a dose of 1 g orally twice daily starting immediately post-transplant. The dosing regimen was adjusted to 500 mg orally four times daily for patients experiencing adverse gastrointestinal effects. For refractory symptoms the dose of mycophenolate mofetil was reduced at the discretion of the treating physician.

Acute rejections were treated with intravenous corticosteroids, rATG, intravenous immunoglobulin, and/or plasmapheresis (the latter two treatments for acute antibody-mediated rejection). Rituximab was also used in cases of refractory acute antibody-mediated rejection. Biopsies were performed for indication and reviewed by a renal pathologist using the Banff classification.

### Exposure and outcome assessment

Patients were grouped according to the timing of the first dose of rATG. Those who received the first dose prior to graft reperfusion (pre- or intra-operatively) were included in the ‘Pre’ group. Patients receiving the first dose of rATG postoperatively were included in the ‘Post’ group.

The primary outcome was the eGFR using the Chronic Kidney Disease Epidemiology Collaboration estimated glomerular filtration rate (CKD-EPI) equation at one year post-transplant [8]. The secondary outcome was the composite endpoint of BPAR, graft loss, or death. All forms of acute rejection (T cell-mediated and antibody-mediated) were included in the composite endpoint. Graft loss was defined as the need to return to chronic dialysis or pre-emptive re-transplant. All deaths occurred prior to graft loss.

### Potential confounders

Recipient, donor, and transplant characteristics, as well as the indication for rATG induction, total rATG dose, starting time of rATG infusion and duration of therapy, were collected through a review of medical charts and our center’s Comprehensive Renal Transplant Research Information System (CoReTRIS) database. Recipient characteristics included age, sex, race, cause of end-stage renal disease (ESRD), body mass index, peak panel reactive antibody (PRA) level, time on dialysis, and re-graft status. Donor characteristics included donor type (deceased versus living), age, sex, and body mass index. Along with induction therapy, transplant characteristics included maintenance immunotherapy, occurrence of DGF (defined as the need for at least one dialysis session within the first week post-transplant) and transplant era (grouped as 2002 to 2004, 2005 to 2007, and 2008 to 2009). All of the above variables were included as potential confounders in our statistical models.

Total rATG dose (adjusted for weight) and indication for induction were also included in all statistical models. Indication categories included high-risk recipient and donor, high-risk recipient, high-risk donor, and low-risk recipient and donor. Categorization was based on the clinical assessment of the attending physician as noted in the medical chart. In addition, recipients were classified as high-risk if any of the following were noted: peak PRA greater than 10%, presence of known donor-specific antibody, or re-graft status. Donors were considered high-risk if they were expanded criteria (ECD) or donation after cardiac death (DCD).

### Statistical analysis

Standard baseline characteristics were compared using parametric (unpaired t-test or one-way analysis of variance (ANOVA)) and non-parametric (Mann-Whitney U test or Kruskal-Wallis test) for continuous variables as appropriate. Proportions were compared using the Fisher’s exact or the chi-square tests. The association of timing and CKD-EPI eGFR at 12 months was evaluated while simultaneously accounting for confounding variables using multiple linear regression models. Changes in eGFR from month 1 to month 12 were also calculated in order to relate the induction timing strategy to the trajectory of eGFR over the first year post-transplant.

Time to the composite endpoint of first BPAR, graft failure, or death was examined using multivariable Cox proportional hazards models. The individual components of the composite endpoint were also evaluated separately. The timing of induction therapy was introduced as an indicator variable with the pre/intra-operative dosing strategy serving as the referent group. Other recipient, donor, and transplant covariates were introduced to adjust for potential confounding. An interaction term was used in our fully adjusted models to determine if donor type significantly modified the association between induction timing and each of the outcomes studied. The assumption of proportionality was graphically examined using log (cumulative hazard) plots and scaled Schöenfeld residuals. No important violations of the proportionality assumption were identified.

All analyses were performed using Stata/MP 11.2 (College Station, TX, USA). A two-sided *P* value of <0.05 was considered statistically significant. Approval for the study was obtained through the Research Ethics Board of the University Health Network.

## Results

A total of 555 KTR received induction therapy with rATG during the 8-year study period. From this cohort, 78 patients were excluded (38 in desensitization protocols, 21 multiple organ recipients, 14 with primary nonfunction, and 5 lacking follow-up data). Of the 477 remaining patients, 124 (26%) had rATG induction initiated prior to graft re-perfusion (Pre), while 353 (74%) started rATG post-operatively (Post). Overall, the median time to beginning the rATG infusion in the Post group was 7.3 hours from the time of leaving the operating room (interquartile range 5.4 to 15.1 hours; n = 268 observations). Median time to starting rATG was greater for those who developed DGF (n = 74) compared to those who did not (n = 194) (9.2 versus 6.6 hours respectively, *P* < 0.001).

Baseline characteristics were similar between groups and most potential confounders were equally distributed (Table 1). Patients in the Post group were more likely to be older, receive a deceased donor transplant, have a shorter cold ischemia time, have a longer length of hospital stay and experience DGF. A significant era effect was observed with more patients receiving the first dose of rATG post-operatively in the later era. Patients in the Post group were also more likely to be on a tacrolimus-based regimen. There were no statistically significant differences between the groups in the other variables that were assessed.

**Table 1:** Recipient, donor, and transplant characteristics

**Baseline characteristics**	**Total (n = 477)**	**Pre (n = 124)**	**Post (n = 353)**	***P *** **value**
**Recipient characteristics**				
Mean recipient age (years)	477	48.5 ± 13.3^a^	51.3 ± 13.1	0.04
Recipient sex				0.14
Male	296	70 (56.5%)	226 (64.0%)	
Female	181	54 (43.6%)	127 (36.0%)	
Race				0.19
Caucasian	289	69 (55.7%)	220 (62.3%)	
Non-Caucasian	188	55 (44.4%)	133 (37.7%)	
Mean height (cm)	477	167.9 ± 11.9	168.1 ± 9.9	0.83
Mean weight (kg)	477	77.7 ± 18.1	75.1 ± 16.3	0.13
Body mass index (kg/m^2^)	477	27.6 ± 5.8	26.5 ± 5.2	0.07
Cause of ESRD				0.58
Diabetes	83	17 (13.7%)	66 (18.7%)	
Glomerulonephritis	171	51 (41.1%)	120 (34.0%)	
Polycystic kidney disease	55	15 (12.1%)	40 (11.3%)	
Hypertension	48	12 (9.7%)	36 (10.2%)	
Other	120	29 (23.4%)	91 (25.8%)	
Transplant number				0.57
First graft	396	105 (84.7%)	291 (82.4%)	
Re-graft	81	19 (15.3%)	62 (17.6%)	
Median PRA (%)^b^	477	15 (0,49)	10 (0,46)	0.23
Median length of stay (days)^b^	477	9 (7,12)	11 (8,16)	0.01
**Donor characteristics**				
Mean donor age (years)	477	45.8 ± 13.9	48.5 ± 14.2	0.05
Donor sex				1.00
Male	250	65 (52.4%)	185 (52.4%)	
Female	227	59 (47.8%)	168 (47.6%)	
Median donor CrCl (mL/min)^b, c^	445 (117/328)^d^	110.6 (92.9, 137.6)	110.5 (91.5, 140.9)	0.88
Mean CIT (min)^e^	244 (38/206)^d^	954.7 ± 314.7	810.7 ± 375.2	0.03
Donor type (%)				0.002
Deceased	312	67 (54.0%)	245 (69.4%)	
Living	165	57 (46.0%)	108 (30.6%)	
DCD kidneys				
No	443	124 (100%)	319 (90.4%)	<0.001
Yes	34	0 (0%)	34 (9.6%)	
**Transplant characteristics**				
Transplant era (%)				<0.001
2002 to 2004	86	31 (25.0%)	55 (15.6%)	
2005 to 2007	186	61 (49.2%)	125 (35.4%)	
2008 to 2009	205	32 (25.8%)	173 (49.0%)	
Delayed graft function				
No	332	99 (79.8%)	233 (66.0%)	0.004
Yes	145	25 (20.2%)	120 (34.0%)	
Maintenance CNI				<0.001
Tacrolimus	360	78 (62.9%)	282 (79.9%)	
Cyclosporine	106	45 (36.3%)	61 (17.3%)	
CNI-free	11	1 (0.8%)	10 (2.8%)	

CIT, cold ischemia time; CNI, calcineurin inhibitor; CrCl, creatinine clearance; DCD, donation after cardiac death; ESRD, end stage renal disease; PRA, panel reactive antibodies.

^a^Plus-minus values are means ± standard deviation.

^b^Median (interquartile range).

^c^Creatinine clearance estimated using the Cockcroft-Gault formula.

^d^Total number assessed (Pre group/Post group).

^e^Deceased donor kidney transplants only.

Table 2 displays the indications for induction therapy and specific treatment characteristics. The most common indication for rATG in the Pre group was high-risk recipient (53.2%), whereas in the Post group it was low-risk recipient with a low-risk donor (40.2%). The mean total and weight-adjusted dose of rATG was significantly higher in the Pre group. The total number of doses administered was similar between the two groups.

**Table 2:** rATG indication and treatment characteristics

	**Pre (n = 124)**	**Post (n = 353)**	***P *** **value**
Indication for rATG			<0.001
High risk recipient and donor	3 (2.4%)	28 (7.9%)	
High risk recipient	66 (53.2%)	112 (31.7%)	
High risk donor	16 (12.9%)	71 (20.1%)	
Low risk recipient and donor	39 (31.5%)	142 (40.2%)	
Mean total dose rATG (mg)	467.7 ± 157.5^a^	379.5 ± 160.0	<0.0001
Mean total weight-adjusted dose rATG (mg/kg)	6.0 ± 1.4	5.1 ± 2.0	<0.0001
Median number rATG doses^b^	5 (4, 6)	5 (4, 6)	0.17

rATG, rabbit antithymocyte globulin.

^a^Plus-minus values are means ± standard deviation.

^b^Median (interquartile range).

Thirteen and 29 patients were excluded from the primary endpoint analysis in the Pre and Post groups respectively due to graft loss, death with function, or loss to follow-up within the first year. Timing of the first dose of rATG failed to demonstrate a statistically significant association with the CKD-EPI eGFR at 12-months (mean CKD-EPI eGFR of 55.0 ± 21.1 versus 56.7 ± 18.2 mL/min in the Post versus Pre groups, *P* = 0.46). Results from the multiple linear regression model are shown in Table 3. When adjusted for recipient, donor, and transplant covariates, the difference between the groups remained small (β = 0.64 (95% CI: -2.82, 4.11), *P* = 0.72). This difference remained similarly non-significant when renal function was assessed using the four-variable MDRD (Modification of Diet in Renal Disease) eGFR equation and after adjustment for the type of calcineurin inhibitor, the use of corticosteroid withdrawal regimens, or for patients receiving only one dose of basiliximab.

**Table 3:** Linear regression models for mean CKD-EPI eGFR at 12 months and mean delta CKD-EPI eGFR at 1 versus 12 months

**Models**	**Mean CKD-EPI eGFR**^**a **^**beta coefficient for timing (95% CI)**	**Mean delta CKD-EPI eGFR**^**a **^**beta coefficient for timing (95% CI)**
1	−1.67 (−6.07, 2.74)	1.52 (−2.06, 5.10)
2	−0.67 (−5.11, 3.77)	0.97 (−2.69, 4.63)
3	1.14 (−2.36, 4.64)	1.62 (−2.16, 5.41)
4	0.88 (−2.51, 4.27)	1.46 (−2.31, 5.23)
5	0.64 (−2.82, 4.11)	1.77 (−2.08, 5.63)

**Model 1** = Timing only.

**Model 2** = Model 1 + indication (high risk recipient and donor, high risk recipient, high risk donor, low risk recipient and donor).

**Model 3** = Model 2 + 1-month recipient eGFR (CKD-EPI) + weight-adjusted total rATG dose + recipient sex + recipient BMI + recipient age + recipient race (Caucasian or non-Caucasian) + cause of ESRD (diabetes or non-diabetes) + time on dialysis + peak PRA + re-graft status.

**Model 4** = Model 3 + donor age + donor sex + donor type (living or deceased).

**Model 5** = Model 4 + transplant era + occurrence of DGF.

BMI, body mass index; CKD-EPI, Chronic Kidney Disease Epidemiology Collaboration; DGF, delayed graft function; eGFR, estimated glomerular filtration rate; ESRD, end stage renal disease; PRA, panel reactive antibodies; rATG, rabbit antithymocyte globulin.

^a^in mL/min.

The difference in eGFR (that is, delta eGFR) from 1 to 12 months was similar across the groups (1.8 ± 16.4 versus 0.3 ± 17.2 mL/min for Post versus Pre groups, *P* = 0.40). In the adjusted model, the difference in delta eGFR for the Post versus Pre groups was not statistically significant (β = 1.77 (95% CI: -2.08, 5.63), *P* = 0.37) (Table 3).

Inclusion of an interaction term in our fully adjusted models revealed that donor type did not significantly modify the association between rATG timing and eGFR (12 months) and delta eGFR (1 to 12 months) (data not shown).

### Composite of BPAR, graft loss or death

**Figure 1:**
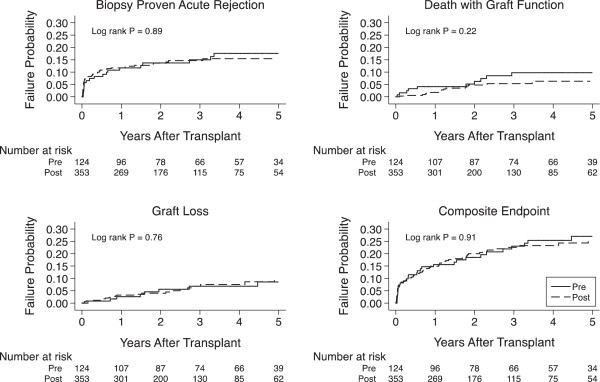
Kaplan-Meier curves for time to biopsy-proven acute rejection, graft loss, death with graft function, and the composite endpoint by the rATG timing group.

The median follow-up time was 3.0 years (interquartile range 1.6 years to 4.9 years). This constituted a total of 1200.1 person-years at risk for the composite outcome. There was no significant difference in time to first BPAR, graft loss, death or the composite across treatment groups as depicted by the Kaplan-Meier failure functions in Figure 1. These findings were confirmed in the multivariable Cox proportional hazards model after adjustment for potential confounders (Table 4). The adjusted hazard ratio for the composite endpoint was 0.94 (95% CI: 0.58, 1.53; *P* = 0.81). Inclusion of an interaction term for timing of induction and donor type in our fully adjusted model showed that donor type did not significantly modify the association between timing of induction and the outcomes of first BPAR, graft loss, death, or the composite (*P* values for interaction = 0.61, 0.27, 0.48, 0.69, respectively). Moreover, adjustment for the type of calcineurin inhibitor at hospital discharge or estimating the treatment effect in a propensity score matched subcohort (85 Pre/Intra versus 85 Post) corroborated the null association of timing and the study outcomes observed in the primary analysis (data not shown).

**Table 4:** Cox model for biopsy-proven acute rejection, graft loss and death (n = 477) over 5-year follow-up

	**Number of events**^**a**^	**Pre (n = 124)**^**b**^	**Post (n = 353)**^**b**^	**Hazard Ratio (95% CI)**^**c**^	***P *** **value**
BPAR	68 (19/49)	17.6%	15.5%	0.90 (0.50, 1.64)	0.73
Graft loss	29 (8/21)	8.6%	10.1%	1.04 (0.41, 2.61)	0.94
Death	26 (10/16)	9.8%	6.3%	0.54 (0.21, 1.39)	0.20
Composite	103 (29/74)	27.0%	25.7%	0.94 (0.58, 1.53)	0.81

^a^Total number of events (Pre group/Post group).

^b^Cumulative probability of the outcome at 5-years using the Kaplan-Meier product limit method.

^c^Predictors in the Cox model include: timing of induction, indication (high risk recipient and donor, high immunologic risk recipient, high risk donor and low immunologic risk recipient), occurrence of DGF, weight-adjusted total rATG dose, recipient age, recipient sex, recipient race (Caucasian or non-Caucasian), cause of ESRD (diabetes or non-diabetes), time on dialysis, peak PRA, re-graft status, recipient BMI, donor age, donor sex, donor type (living or deceased), and transplant era (2002 to 2004, 2005 to 2007 and 2008 to 2009).

BMI, body mass index; BPAR, biopsy-proven acute rejection; CI, confidence interval; DGF, delayed graft function; ESRD, end stage renal disease; PRA, panel reactive antibodies; rATG, rabbit antithymocyte globulin.

## Discussion

Timing of the first dose of rATG induction prior to graft reperfusion had no significant impact on renal function at one year when compared to post-operative administration in this retrospective cohort study of adult KTR. In both the unadjusted and adjusted linear regression models, this difference remained nonsignificant. The CKD-EPI equation was used to estimate renal function as it has recently been shown to offer better performance when compared to the MDRD study equation in KTR [8]. Furthermore, postoperative administration of rATG induction had no discernable effect on time to first BPAR, graft loss, death or the composite when evaluated over the 5-year follow-up period. These findings were confirmed in multivariable Cox regression models.

Notably, patients in the Post group were more likely to be older, recipients of deceased donor organs, transplanted later in the study period, and had longer lengths of stay in hospital. This is a reflection of time trends in the population transplanted at our center, with recipients of greater medical complexity and an increasing use of ECD and DCD donor organs in the more recent era. Lower dosing of rATG in the Post group reflects practice changes over time. In 2007, we instituted a new immunosuppression protocol for low-risk patients that included low dose rATG (3 mg/kg) for induction. In recent years there has also been a trend towards reducing the total dose of rATG induction for high-risk patients. However, when controlling for these variables in the adjusted model we observed no difference in outcome by the two timing strategies. In fact, the higher prevalence of negative risk factors and lower rATG dosing in the Post group would suggest that the presence of residual confounding bias would lead to worse outcomes for the Post group. This increases the robustness of our inferences.

A number of studies have shown the efficacy of postoperative rATG induction in reducing acute rejection rates [2-4], however others have suggested that intra-operative infusion may be preferable [5,9-12]. In a prospective, randomized, controlled trial of intra-operative versus post-operative rATG among 58 KTR, Goggins *et al.* demonstrated that the incidence of DGF is reduced with administration prior to cross-clamp removal [6]. Ischemia has been shown to cause endothelial activation and promotion of leukocyte adhesion and trafficking into the allograft, which in turn leads to expression of co-stimulatory molecules, cytokine release, and lymphocyte activation [13]. Since rATG is comprised of antibodies to many of these ischemic-reperfusion injury mediators, it may act to attenuate this response [14,15]. While the intra-operative group had a lower mean serum creatinine at 14 days post-transplant, this early difference in renal function was not statistically significant by day 30 [6].

From a practice standpoint, operational factors favor postoperative administration of rATG. Preparation of this drug involves dose calculation, reconstitution of multiple medication vials, dilution in an appropriate volume, and limited stability that precludes advance admixing. Pre-medication is required, and the infusion must be run through a high-flow vein using an inline filter over a minimum of 6 hours with close monitoring [1]. While some centers have been able to address these issues through strategies such as order sets, protocols, education, and involvement of the clinical pharmacist, time between patient arrival and transfer to the operating room is often insufficient to allow for pre-operative dosing and physical transport of the patient with rATG infusing may present a challenge.

Intra-operative dosing also introduces safety risks such as erroneous administration technique, which has consistently been one of the most harmful types of medication errors in health systems reporting [16]. The typical process for medication administration in the operating room is dramatically abbreviated and lacks the usual safeguards that exist in other areas of the hospital [7]. In a Canadian survey of 687 anesthesiologists, 85% report a drug error or a near miss in clinical practice [17]. In a New Zealand study, 12.5% of practitioners surveyed reported that they were aware of harm caused to a patient as a result of a drug administration error [18]. In mid-2007, computerized prescriber order entry was instituted on the inpatient wards at our hospital. This change introduced further complexities to the peri-operative workflow since the operating room retained a paper-based process. Due to the occurrence of a number of severe intra-operative medication errors involving rATG, a practice change was instituted in late 2007 to have rATG administered in the immediate post-operative setting. This allowed for management by experienced staff, simplified workflow, and has since eliminated serious medication errors associated with rATG at our center.

While intra-operative administration of rATG may have a favorable impact on short-term outcomes, there is limited literature to show that a benefit is maintained over time. Such short-term outcomes are certainly relevant, however induction therapy may also have more durable effects that are important to examine. Potential long-term benefits of rATG induction may include improved graft survival by decreasing acute rejection rates and limiting exposure to calcineurin inhibitors in the early post-transplant period. In our study, we observed no differences by timing of rATG induction on clinically meaningful endpoints including eGFR, BPAR, graft loss, or death over the follow-up period.

Our study addresses a clear gap in the literature. The single previous report evaluating timing of rATG induction in KTR involved a small number of patients with a follow-up period of only 30 days. With the shift in clinical practice at our institution we had a unique opportunity to examine the impact of rATG induction timing. The present study is the largest to date and has a median duration of follow-up of 3 years. The use of multivariable regression modeling to adjust for potential confounders strengthens our analysis of timing of induction therapy as it relates to meaningful clinical endpoints. Despite the shift in the later era towards post-operative administration, there remained a significant degree of overlap between pre- versus post-operative administration during the 8-year study period, with good representation of both high and low risk patients in both Pre and Post groups across all transplant eras.

There are limitations to our study that deserve mention. First, this was not a randomized controlled trial, and therefore the possibility of residual confounding exists. To reduce this risk, data were retrieved through a systematic chart review, all variables were defined *a priori,* and multivariable modeling was used to account for differences in baseline characteristics between the groups. Moreover, an assessment of the effectiveness (versus efficacy) of rATG timing in the real world setting is best estimated using a clinical cohort. Second, there may have been a selection bias related to timing since, following implementation of the practice change, 23% of patients in the later era still received rATG pre-operatively. It is possible that some of these cases may have had higher risk features that were not captured in our baseline analysis and may have been pre-disposed to worse outcomes. Notably, it is routine practice in our center to admit patients for living donor kidney transplantation on the night before surgery which allows adequate time for pre-operative rATG administration. This likely accounts for the majority of patients in the Pre group in the later era. Third, since rATG was often prescribed in response to the development of DGF post-operatively, it was not possible to analyze the impact of rATG timing on the incidence of DGF without significant confounding by indication. However, the occurrence of DGF in the early postoperative period was included as a confounder in our statistical models. Fourth, because this study was conducted in a single institution, the findings may not be entirely generalizable to other settings. Finally, although this is the largest study to date on the issue of induction timing and kidney transplant outcomes, it may have been underpowered to rule out small treatment effects.

## Conclusions

The results of this retrospective cohort study of KTR suggest that pre/intra-operative versus postoperative administration of rATG induction has similar implications for renal function at one year post-transplant. Moreover, the risk of developing clinically relevant endpoints over a 5-year follow-up period was comparable across rATG timing groups. While our results do not definitively rule out a small but meaningful benefit of pre/intra-operative administration of rATG, we believe our findings open the door to explore more flexible timing strategies for the first dose of rATG induction therapy in kidney transplantation.

## Abbreviations

BPAR: Biopsy-proven acute rejection; CKD-EPI: Chronic Kidney Disease Epidemiology Collaboration; DCD: Donation after cardiac death; DGF: Delayed graft function; ECD: Extended criteria donor; eGFR: estimated glomerular filtration rate; ESRD: End-stage renal disease; KTR: Kidney transplant recipients; MDRD: Modification of diet in renal disease; PRA: Panel reactive antibodies; rATG: rabbit antithymocyte globulin.

## Competing interests

The authors of this manuscript declare that they have no competing interests. The study sponsor (Genzyme Canada Inc.) played no role in any aspect of the research design, conduct, data analysis, manuscript preparation or submission.

## Authors’ contributions

JJH, BH and SJK participated in designing the study, performing the research, analyzing the data and writing and reviewing the manuscript. YL participated in analyzing the data and reviewing the manuscript. OF participated in performing the research, analyzing the data and reviewing the manuscript. All authors read and approved the final manuscript.

## Authors’ information

JJH (BScPharm, MSc) is the Pharmacy Clinical Site Leader for the Multi-Organ Transplant Program and the Department of Pharmacy Services, Toronto General Hospital, University Health Network. She is also Assistant Professor (Status) with the Leslie Dan Faculty of Pharmacy, University of Toronto, Toronto, Ontario. BH (BScPharm, MSc) is a Staff Pharmacist with the Multi-Organ Transplant Program and the Department of Pharmacy Services, Toronto General Hospital, University Health Network. He is also Assistant Professor (Status) with the Leslie Dan Faculty of Pharmacy, University of Toronto, Toronto, Ontario. YL (MSc) is a Statistician with the Kidney Transplant Program and Division of Nephrology, Toronto General Hospital, University of Toronto, Toronto, Ontario. OF (dipHSM, MPH, MEd, CHE, DHA (c)) is the Research Manager for the Kidney Transplant Program and Division of Nephrology, Toronto General Hospital, University Health Network, Toronto, Ontario. SJK (MD, PhD, MHS, FRCPC) is a Transplant Nephrologist with the Kidney Transplant Program, Toronto General Hospital, University Health Network and the Division of Nephrology, Toronto General Hospital and St Michael’s Hospital, University of Toronto, Toronto, Ontario.
